# Comparison of Methods for Testing Mismatch Repair Status in Endometrial Cancer

**DOI:** 10.3390/ijms241914468

**Published:** 2023-09-23

**Authors:** Marta Mendiola, Victoria Heredia-Soto, Ignacio Ruz-Caracuel, Amparo Baillo, Jorge Luis Ramon-Patino, Francisco Javier Escudero, Maria Miguel, Alberto Pelaez-Garcia, Alicia Hernandez, Jaime Feliu, David Hardisson, Andres Redondo

**Affiliations:** 1 Molecular Pathology and Therapeutic Targets Group, Hospital La Paz Institute for Health Research (IdiPAZ), 28046 Madrid, Spain; mmiguel17@gmail.com (M.M.); alberto.pelaez@idipaz.es (A.P.-G.); david.hardisson@salud.madrid.org (D.H.); 2 Center for Biomedical Research in the Cancer Network (CIBERONC), Instituto de Salud Carlos III, 28029 Madrid, Spain; vic_heredia@yahoo.es (V.H.-S.); jaimefeliu@hotmail.com (J.F.); 3 Translational Oncology Research Laboratory, Hospital La Paz Institute for Health Research (IdiPAZ), 28046 Madrid, Spain; 4 Department of Pathology, La Paz University Hospital, 28046 Madrid, Spain; 5 Mathematics Department, Autonomous University of Madrid, 28049 Madrid, Spain; amparo.baillo@uam.es; 6 Department of Medical Oncology, La Paz University Hospital, 28046 Madrid, Spain; jorge.ramon@startmadrid.com; 7 Department of Obstetrics and Gynecology, La Paz University Hospital, 28046 Madrid, Spain; aliciahernandezg@gmail.com; 8 Faculty of Medicine, Autonomous University of Madrid, 28046 Madrid, Spain; 9 Cátedra UAM-ANGEM, Faculty of Medicine, Autonomous University of Madrid, 28046 Madrid, Spain

**Keywords:** endometrial carcinoma, methylation, mismatch repair deficiency, microsatellite instability

## Abstract

Approximately 20–30% of endometrial carcinomas (EC) are characterized by mismatch repair (MMR) deficiency (dMMR) or microsatellite instability (MSI), and their testing has become part of the routine diagnosis. The aim of this study was to establish and compare the MMR status using various approaches. Immunohistochemistry (IHC), PCR-based MSI, and the detection of defects in the four key MMR genes (MLH1, PMS2, MSH2, and MSH6) via methylation-specific multiplex ligation-dependent probe amplification (MLPA) and targeted next-generation sequencing (NGS) were performed. MSH3 expression was also evaluated. A set of 126 early-stage EC samples were analyzed, 53.2% of which were dMMR and 46.8% of which were proficient MMR (pMMR) as determined using IHC, whereas 69.3% were classified as microsatellite stable, while 8.8% and 21.9% were classified MSI-low (MSI-L) and MSI-high (MSI-H), respectively. In total, 44.3% of the samples showed genetic or epigenetic alterations in one or more genes; MLH1 promoter methylation was the most common event. Although acceptable concordance was observed, there were overall discrepancies between the three testing approaches, mainly associated with the dMMR group. IHC had a better correlation with MMR genomic status than the MSI status determined using PCR. Further studies are needed to establish solid conclusions regarding the best MMR assessment technique for EC.

## 1. Introduction

Mismatch repair (MMR) is one of the main DNA repair pathways that essentially correct polymerase errors generated during replication. MMR deficiency is triggered via the loss of function of at least one of its components, mainly MLH1, PMS2, MSH2, and MSH6. This loss can be promoted by various mechanisms, such as pathogenic mutations on the gene coding sequence and promoter methylation mechanisms [1,2].

Gene loss of function is linked to a decrease in the corresponding protein expression, which can be identified via immunohistochemistry (IHC). The loss of one or more markers is referred to as deficient MMR (dMMR), while the retention of expression of these four markers is known as proficient MMR (pMMR). Individual losses can occur, but dual losses are frequent due to the nature of the heterodimeric MMR protein complexes (MLH1/PMS2 and MSH2/MSH6). Previous studies have concluded that a 2-IHC-marker approach would be sufficient to detect dMMR, while other studies have suggested that additional MMR molecules, such as MSH3, should be explored [3,4].

Microsatellites (MS) are an alternate surrogate marker for MMR status. These short highly polymorphic sequences have high mutational rates and are profoundly affected by defects in the MMR pathway [5,6]. Their repetitive structure makes them susceptible to accumulating deletions and expansions during replication, a phenomenon known as MS instability (MSI). An alteration in length is considered an unrepaired mistake and is usually linked to dMMR. In contrast, MS stability (MSS) is considered to have intact MMR machinery and is therefore associated with pMMR [7]. Other approaches can be used to directly identify the alterations in the MMR genes. Next-generation sequencing (NGS) targeted panels can detect mutations, while methylation-specific multiplex probe amplification (MS-MLPA) can be used to detect promoter methylation events [8,9,10]. MS-MLPA is based on the hybridization of probes that recognize specific sequences in DNA containing restriction sites for a methylation-sensitive endonuclease. After digestion, amplification, and fragment analysis, the signal appears only for methylated samples [11].

The widely adopted procedures for MSI evaluation rely on PCR and Sanger capillary electrophoresis sequencing methods. The most used panels are the Bethesda one, which analyzes two mononucleotides (BAT-25 and BAT-26) and three dinucleotides (D5S346, D2S123, and D17S250) repeats, and alternatively, the one covering five mononucleotide repeats (BAT-25, BAT-26, NR-21, NR-24, and NR-27), included in the Promega MSI Analysis version 1.2 [12,13]. Additional PCR-based kits based on these MS or alternative panels, as well as other options based on PCR and high-resolution melting or chip microfluidic electrophoresis, are now available as alternative MSI analysis options [14,15,16].

Approximately 20–30% of cases of endometrial cancer (EC) cases are characterized by dMMR/MSI [8]. Of those, 80–90% are associated with sporadic disease and are caused mainly by hypermethylation of the MLH1 promoter, as is the case for colorectal cancer (CRC) [17,18]. The remaining 10–20% of dMMR cases are related to hereditary Lynch syndrome due to pathogenic germline mutations in MLH1, PMS2, MSH2, and/or MHS6 [19,20]. MMR/MSI testing is now recommended in EC as a diagnostic tool in cases with ambiguous morphology, to screen for Lynch syndrome, and for the molecular characterization of this cancer, harboring prognostic and therapeutic implications [21]. 

In 2013, The Cancer Genome Atlas (TCGA) Consortium published a comprehensive EC genomic characterization system, establishing four molecular subtypes [8], one of which is characterized by dMMR/MSI and associated with an intermediate risk of relapse [22].

This study demonstrates that molecular classification has an impact on prognosis, as other clinicopathological factors do, leading to their inclusion for risk assessment in the guidelines for EC management published in 2020 by the European Society of Gynaecological Oncology, the European Society for Radiotherapy and Oncology, and the European Society of Pathology [23]. Improving our understanding of EC is a significant challenge, especially with regard to treatment selection. Recently, some clinical trials have shown the importance of MSI/dMMR as a predictive biomarker for immunotherapy in advanced stages [24,25,26,27]. The aim of this study was to compare the detection of MMR defects via IHC, MSI status via PCR, and MMR genomic status via MS-MLPA and targeted NGS to investigate if they are diagnostically equivalent in EC.

## 2. Results

### 2.1. Sample Selection and Initial Screening

A total of 289 cases of early-stage EC were screened for dMMR status via IHC on tissue microarrays (TMAs) assessing four markers: MLH1, PMS2, MSH2,and MSH6. Thirty cases could not be classified by TMA evaluation, and IHC was performed in complete tissue sections. Ultimately, two cases (0.7%) could not be assessed.

**Figure 1:**
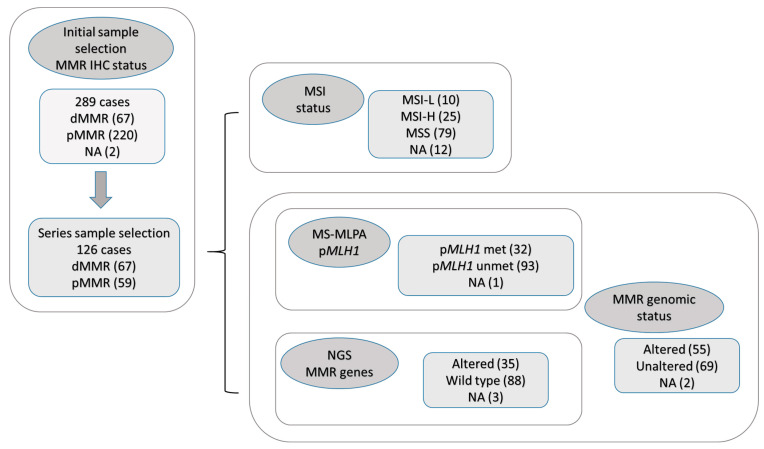
Study flowchart. MMR, mismatch repair; IHC, immunohistochemistry; dMMR, deficient MMR; pMMR, proficient MMR; MSI, microsatellite instability; MSI-L, MSI—low; MSI-H, MSI—high; MSS, microsatellite stable; NA: not available; MS-MLPA: methylation-specific multiplex ligation probe amplification, p*MLH1*: *MLH1* promoter; met:Methylated, unmet:unmethylated; NGS: next generation sequencing.

The loss of expression in any MMR protein (dMMR) was observed in 67 cases (23.2% of the global series). Another 59 pMMR cases with no protein loss were randomly selected as the control group. This study ultimately included 126 cases. Figure 1 shows the complete study flowchart and group distributions.

### 2.2. Descriptive Results

#### 2.2.1. MMR Loss of Expression Based on IHC

**Figure 2:**
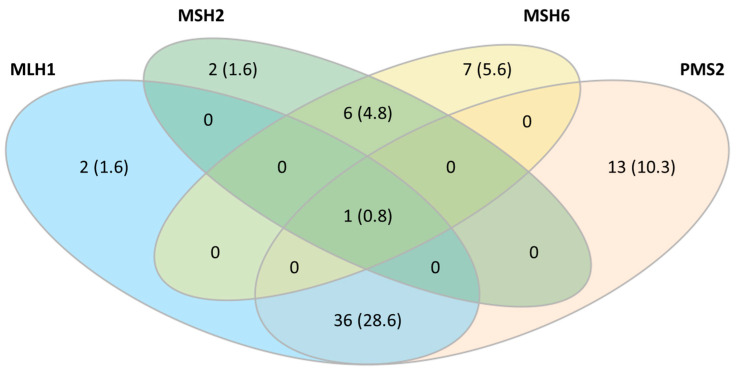
Venn diagram for immunohistochemistry category distribution.

Figure 2 summarizes the protein expression patterns in the dMMR group. Dual loss of MLH1/PMS2 was the most frequent pattern and was observed in 28.6% of cases.

MSH3 expression was also assessed using IHC, with a valid result for 114 cases. Loss of MSH3 expression was detected in 15 (13.2%) cases, with concomitant loss of MSH2 in 4 cases and concomitant loss of MSH6 in 5 cases.

#### 2.2.2. MSI Status Based on MS PCR

MS status was assessed with the Promega 1.2 MSI Detection kit, which was not evaluable in 12 of the 126 cases (9.5%) due to insufficient DNA quality; these 12 cases were excluded from further calculations. The MS status distribution was as follows: 25 (21.9%) cases MSI-high (MSI-H), 10 (8.8%) MSI-low (MSI-L), and 79 (69.3%) MSS (Figure 1). Supplementary Table S1 provides a detailed distribution of the observed MS alterations. The MSI pattern was observed in the following order: MONO-27, BAT26, BAT25, NR24, and NR21. The most representative MSI pattern was one or two unstable MS, which was observed in 15.8% of the cases. The current standard recommends including MSI-L in the MSS category [17]. We explored this classical distribution while including the alternative “pure MSS” and “all MSI” (MSI-L and MSI-H).

#### 2.2.3. MMR Genomic Status

Alterations in the coding sequence of *MLH1*, *PMS2*, *MSH2* and *MSH6* were confirmed using NGS, and *MLH1* promoter hypermethylation was confirmed using MS-MLPA. In total, 2 (1.6%) of the 126 cases were not assessable due to technical issues and were excluded from further calculations. Figure 1 and Supplementary Table S2 summarize the distribution of gene alterations. Fifty five (44.3%) of the 124 cases showed genomic alterations, the most frequent of which was the *MLH1* gene, mainly via promoter hypermethylation (22.6% of 124 cases).

### 2.3. Comparisons

#### 2.3.1. IHC: Consideration of Two Markers and Inclusion of MSH3, versus Four Markers

We employed Fisher’s exact test of independence to evaluate the concordance of MLH1 loss with PMS2 loss and MSH2 loss with MSH6 loss, obtaining significant *p*-values (2.2 × 10^−16^ and 1.3 × 10^−6^, respectively), and suggesting a strong association between the doublets. Sixty-seven cases were classified as dMMR based on four-marker IHC compared to sixty-three based on two-marker (PMS2 and MSH6) IHC. Therefore, the two-marker approach resulted in a misclassification of 3.2% of cases in the study population. 

Fisher’s exact test also demonstrated an association between MSH3 and both MSH2 and MSH6 (*p*-values of 1.3 × 10^−6^ and 0.013, respectively). When PMS2, MSH2, and MSH3 were employed, a misclassification of 3.5% was observed.

#### 2.3.2. MMR Genomic Status Compared with IHC Results

This study considered IHC the standard for identifying dMMR cases and compared IHC with the results of MMR genomic status. Forty-six of the 67 dMMR cases (68.7%) showed genetic or epigenetic alterations in one or more genes, whereas 48 of the 57 pMMR cases (84.2%) showed no genomic alteration (Table 1a). The global correlation between IHC status and the genomic results was 0.63 (Fisher’s exact test *p*-value, 1.0 × 10^−8^). 

One-by-one markers and both tandems (MLH1/PMS2 and MSH2/MSH6) were analyzed in cases with available data using both techniques (Supplementary Table S3). 

#### 2.3.3. MS PCR-Based Status Compared with IHC Results

The distribution of the 63 dMMR cases was as follows: 34 (53.9%) cases were MSS, 7 (11.1%) MSI-L, and 22 (34.9%) MSI-H. For the 51 pMMR cases, 45 (88.2%) were MSS, 3 (4.8%) MSI-L, and 3 (4.8%) MSI-H (Table 1b). 

The overall correlation with IHC increased slightly when the alternative MSI approach was considered (36.9%) over the classical one (34.9%), although both values were low. The *p*-value for Fisher’s exact test of independence between the two IHC subgroups and the three MS PCR subgroups was significant (*p* = 0.0001). Supplementary Table S4 summarizes the detailed distribution among the IHC markers and MS status with the two classifications. The results showed acceptable concordance between MLH1, PMS2, and the doublet; however, the proportion of MSS in the protein loss group was high (39% for MLH1 and 51% for PMS2), leading to a low correlation. The results had low statistical significance, with non-significant *p*-values in Fisher’s exact test in the case of MSH2, MSH6, and their doublet in both MS classifications.

#### 2.3.4. MSI PCR-Based Status Compared with MMR Genomic Status

Of the 63 cases with no genomic alterations, 4 were classified as MSI: 3 (4.8%) as MSI-L and 1 (1.6%) as MSI-H. However, 18 (36.73%) of the 49 cases with an alteration in a MMR gene sequence were classified as MSS (Table 1c). 

The overall correlation increased when the alternative MS classification was applied over the classical classification (60.91% vs. 56.46%, respectively). Supplementary Table S5 summarizes the detailed distribution of the MMR genomic status and the two MS classifications. The results showed acceptable concordance (>80%) between MLH1 and PMS2 alterations and MSI status for the classical MS classification. The only significant correlation between MS PCR status and MMR genomic status corresponded to MLH1. The concordance and correlation were higher when the classical MS classification was applied.

#### 2.3.5. Overall Comparisons

The sensitivity, specificity, and overall proportion of correct classifications were analyzed (Table 2). Taking IHC as the reference, the proportion of cases correctly classified by MMR genomic status was higher (75.8%) than by PCR (61.4% and 64.9% for the classical and alternative classifications, respectively). IHC was, therefore, a better overall predictor of MMR gene status than of MS PCR class, as well as showing a reasonable trade-off between sensitivity and specificity. The sensitivity of IHC dMMR for predicting PCR MSI-H was the highest (88.0%) while having the lowest specificity (53.9%). A better balance between sensitivity and specificity (82.9% and 57.0%, respectively), as well as a higher proportion of correct classifications (64.9% vs. 61.4%), was achieved when MS was split into pure MSS and MSI (MSI-L + MSI-H) categories. 

When genomic status was considered the reference, the proportion of correct classifications via PCR was higher than IHC (75.8%). Once again, this proportion was higher with the alternative MS classification (80.4%) than with the classical one (76.8%), although the sensibility of the former was lower (88.6% vs. 96.0%).

Overall, IHC and PCR displayed a lower degree of mutual dependence and ability to predict each other than IHC and gene status or gene status and PCR. This situation was also reflected in the correlations and concordance proportions separated by markers (see Supplementary Tables S4 and S5), where the highest values were achieved via genomic MLH1. In summary, there was acceptable concordance between the MSS and pMMR categories but a high proportion of discrepancies between MSI and dMMR. The same tendency was observed between MSS and no sequence alteration, as well as between MSI and sequence alterations in the MMR genes. Additionally, the correlation increased when the MSI categories were split into pure MSS and all MSI (MSI-L and MSI-H), suggesting that the MSI categories should be reconsidered in EC.

## 3. Discussion

EC is one of the types of cancer most commonly associated with the dMMR/MSI-H phenotype. There is currently a strong recommendation to assess the MMR status in EC, and the main guidelines recommend IHC for analyzing this biomarker [23]. The importance of accurately defining the dMMR/MSI-H status is tied to the presence of Lynch syndrome, which entails an increased risk of developing associated tumors in patients and their families. In addition, this biomarker defines one of the intermediate risk groups in the latest proposed EC classifications due to the prognostic implications. The dMMR/MSI-H status also helps predict the benefit of adjuvant therapies and immunotherapy in advanced stages [24,25,26,27,28].

MMR detection techniques have been developed in the CRC setting, and published studies have shown that EC might not constitute the same scenario. In this study, we employed various approaches to determine MMR status: IHC detection of MMR protein expression, PCR detection of MS status (based on five MS, using a commercially available kit [Promega 1.2]), and detection of genomic status, analyzing the four MMR genes via NGS and the identification of methylation defects on the MLH1 promoter via MS-MLPA. The results of these three approaches and their comparisons from a single-center early-stage EC series are presented.

Compared with other approaches, IHC is widely available and not expensive. The presence of stained non-neoplastic cells in the sample represents an internal positive control that easily identifies false negative results. Another advantage of IHC over PCR is that the loss-of-expression pattern provides information on the particular altered gene, guiding later sequencing to rule out Lynch syndrome.

Although the expression of the four markers (MLH1, PMS2, MSH6, and MSH2) is a reliable method, the use of only two markers (PMS2 and MSH6) has been proposed as a more cost-effective alternative for MMR testing [3,29,30]. MMR proteins act as heterodimers (MLH1-PMS2 and MSH2-MSH6). PMS2 and MSH6 are only stable within the complexes, and their expression is lost when those are not present. PMS2 loss can, therefore, potentially identify all cases with PMS2 loss alone and with MLH1-PMS2 dual loss, while MSH6 loss would detect cases with isolated MSH6 loss and combined MSH2-MSH6 loss. The main disadvantages of this simplified method are that (1) a second IHC round with MLH1 and MSH2 should be performed for dMMR cases if the specific gene needs to be identified, and (2) cases with isolated loss of MLH1 and MSH2 are potentially missed. These cases represent 0–3% of the published series and of a recent meta-analysis [31]. The two-marker approach misidentified four cases (3%) in our series. Although two-marker IHC testing might be acceptable for the initial screening of dMMR, considering that MMR status can identify the best candidates for immunotherapy, more studies are needed to establish solid conclusions.

Other molecules also participate in the MMR pathway, although there is scarce available data regarding their pathogenicity. MSH3 and MSH6 have been described as partially redundant for MMR, acting in conjunction with MSH2 [32]. 

MS analysis via PCR is an alternative to IHC for identifying MMR defects but is more laborious than IHC because it requires a more specific tissue selection and DNA extraction, usually from tumors and non-neoplastic tissues. With this PCR approach, the specific altered gene is not identified in order to discard Lynch syndrome.

MSI is determined based on the pattern observed in a subset of MS and requires expertise for its interpretation. As stated in the introduction, new approaches recently evolved, with differences in their performance, would merit further evaluation in EC; the most traditional and widely used for decades have been the Bethesda panel (composed of two mononucleotide and three dinucleotide loci) and other commercially available panels consisting of five mononucleotides (like Promega 1.2) [14,15,16].

A number of authors have suggested combining IHC and PCR techniques to achieve reliable results, while others have compared the two approaches. An acceptable concordance of approximately 90% between IHC and PCR has been reported in EC studies [33,34,35,36]. Discrepancies between studies have been attributed to the fact that MS panels have been originally selected and adjusted for CRC associated with Lynch syndrome, and the instability pattern could be related to the tumor type [37]. Indeed, EC displays minimal MS shifts, with a mean shift of 1–3 nucleotides, compared to the six reported for CRC [38].

Our study’s overall concordance between IHC and PCR is lower than that reported in previous studies, which could be partly explained by differences in design. Most published studies were performed on an unselected EC population (usually consisting of 70–80% of pMMR cases), whereas our study included a balanced number of cases in both MMR groups. Studies performed on unselected cases reported only 2–8% of discrepancies in the overall comparisons [33,39]. Given that most of the discrepancies we detected occurred in the dMMR group, these could be minimized in an overall concordance analyzed in unselected studies with a high proportion of pMMR cases. Although most of the published studies focused on overall concordance, a number of them also explored the results in the MMR subgroups. Stello et al. observed a high overall concordance between IHC and PCR but stated that most of the discordant cases involved loss of MMR protein expression and an MSS/MSI-L phenotype [21]. Ferguson et al. made comparisons in the MMR subgroups and reported that 21% of the IHC dMMR tumors were MSS via PCR [40]. In enriched dMMR population studies with an overrepresentation of dMMR cases, discrepancies of more than 10% have been reported between IHC and PCR, which are in line with our results [36,41].

A number of authors have reported an association between the pMMR pattern determined using IHC and MSI-L determined using PCR. MSI-L tumors present clinicopathologic features similar to MSS tumors in CRC and are frequently grouped as MSS [42,43]. In our series, however, a higher proportion of MSI-L cases were classified as dMMR via IHC, and all but one showed MMR genetic alterations. These conflicting results require further research to be clarified because they might raise the possibility of combining MSI-L and MSI-H in the same category, at least for EC. We studied the three categories separately and assessed the correlation for an alternative option: the pure MSS category when all MS show a stable pattern and MSI when any of the MS are unstable. A slight increase in the correlation between categories for MSI and MMR via IHC was observed with this option. The same tendency occurs when comparing MS via PCR to the MMR genomic status. Our results show that MSI-L in EC could be informative by itself, but further studies are needed to confirm this conclusion.

Targeted sequencing is the most complex and expensive of the three techniques and depends on the availability of sequencing facilities, which are not always affordable for routine practice. For hypermethylation studies, MLPA-based methods are an option. Both techniques help identify the specific gene alteration and are usually employed to rule out or confirm Lynch syndrome more than for dMMR phenotype screening.

There are fewer published studies that have included genetic or epigenetic alterations, and there is no standardization regarding the panel of choice that might include gene regions with different coverage. Introns are usually not sequenced and could include mutations. Promoter methylation can be also prompted not directly but as a consequence of other alterations, such as the EPCAM gene deletions. These cases, which are related to Lynch syndrome, seem, however, to be more frequently associated with CRC [44,45].

Our study’s design has certain limitations and strengths. The single-center retrospective design could be considered a limitation but could also be a strength, given that the MMR/MSI status was determined in a single center and evaluated by the same experts, an important consideration when comparing results. We employed two of the most widely used approaches for analyzing MMR status (IHC and PCR) while simultaneously obtaining the MMR gene alterations status. This study’s design included balanced groups, where dMMR constitutes 53% of the series instead of 20–30% of real-world EC cases, making it possible to highlight the discrepancies between the techniques in assessing the dMMR/MSI-H phenotype.

Identifying the MMR status is important for selecting patients who will derive the greatest benefit from immunotherapy. Using IHC, the GARNET study showed a higher response rate in the dMMR population than in the pMMR population [27]. The RUBY and NRG-GY018 phase III trials have recently shown a significant progression-free survival benefit when adding an anti-PD1 to first-line chemotherapy, a benefit that was greater for the dMMR population [24,25]. Given that there are also pMMR patients who can benefit from immunotherapy, further studies are needed to improve the accuracy of the current biomarker (MMR status) and to identify additional biomarkers to improve the identification of patients who will respond to checkpoint inhibitors.

## 4. Materials and Methods

### 4.1. Patients and Samples

Patients with early-stage EC and available tissue who were diagnosed between 2003 and 2015, as recorded in La Paz University Hospital archives, were screened. 

IHC was employed as the reference technique to establish two patient groups based on the MMR biomarker. The first group consisted of all patients with dMMR. A comparable number of patients from the pMMR population were then randomly selected to form the control group. 

This study was approved by the local ethics committee (HULP#PI3778) and was conducted in accordance with the ethical standards of the Declaration of Helsinki of the World Medical Association.

### 4.2. Immunohistochemistry

Representative tumor areas were selected by experienced pathologists, evaluated in hematoxylin-and-eosin (H&E) sections, and employed for TMA inclusion selection, as previously described [46]. MMR status was determined using IHC with specific primary antibodies for MLH1 (clone ES05, #IR079), PMS2 (clone EP51, #IR087), MSH2 (clone FE11, #IR085), and MSH6 (clone EP49, #IR086), and with the Envision detection kit (all from Agilent Technologies, Santa Clara, CA, USA). Entire sections from the original tissue block were examined when the case was not assessable in the TMA.

The presence of dMMR was considered when the complete loss of nuclear expression of one or more markers in tumor cells was observed. Normal stromal tissue cells demonstrating nuclear staining served as an internal positive control [47]. Additionally, MSH3 expression was analyzed with a primary specific antibody (clone RM405, #275928, Abcam, Cambridge, UK).

### 4.3. DNA Extraction

Selected tissue blocks with enriched tumor and non-tumor areas were employed for DNA extraction with the QIAamp DNA FFPE Tissue Kit (Qiagen GmbH, Hilden, Germany) and fluorometrically quantified using Qubit (Thermo Fisher, Waltham, MA, USA). All subsequent analysis was performed blinded to the IHC result.

### 4.4. MSI Testing

For the MSI testing, we employed the Promega 1.2 test kit (#MD1641, Promega, Madison, WI, USA), which contains fluorescently labeled primers for co-amplification of 7 markers: 5 mononucleotides (BAT-25, BAT-26, NR-21, NR-24, and MONO-27) for MSI, and 2 pentanucleotides (Penta C and Penta D) for control purposes. For interpretation, MSI in only one marker was designated as low (MSI-L), while MSI in two or more markers was designated as high (MSI-H). When no alterations were observed, the case was designated as stable (MSS).

### 4.5. Next-Generation Sequencing

Targeted sequencing was performed in the *MLH1*, *PMS2*, *MSH2*, and *MSH6* genes at Fundación Parque Científico de Madrid (Madrid, Spain). Samples were enriched for the specific genes using the custom panel PDG518v2 (Paragon Genomics, Hayward, CA, USA), which consisted of 179 amplicons and covered 96.35% of the target regions. Libraries were sequenced in MiSeq (Illumina, San Diego, CA, USA) with the paired-end 2 × 150 sequencing format. 

After sequencing, PRINSEQ was employed to remove reads shorter than 70 nucleotides and to filter out possible sequences [48]. The alignment rate of the reads (using the Bowtie alignment program) was then evaluated and found to be >99% [49], with a mean depth of approximately 1.000×. Variants were annotated using automated pipelines, and potential pathogenic variants were identified using the VarSome search engine (Saphetor, Lausanne, Switzerland) [50]. Further validation was performed via manual review using Integrative Genomics Viewer (IGV) software version 2.4.10 [51]. Only described pathogenic mutations were recorded.

### 4.6. MLH1 Promoter Hypermethylation

The MS-MLPA ME011 kit (MRC Holland, Amsterdam, The Netherlands) was employed to set *MLH1* promoter CpG methylation islands. The kit includes probe pairs covering different promoter regions (A to D) and intron 167.

### 4.7. Statistical Analysis

Contingency tables were computed for all pairs of categorical variables of interest (specifically, the IHC markers, the NGS status, and the MSS/MSI-L/MSI-H classes). Each of these tables was employed to perform Fisher’s exact test of independence to check for associations between the variables. Phi (correlation) coefficients and concordance proportions were computed for all pairs of dichotomous variables to describe and detect pairwise dependence. Logistic regressions were fitted to study the ability of marker groups to classify into two statuses to decide which IHC marker best discriminates between the two possible NGS results. The diagnostic performance of these regressions was quantified via the overall proportion of correct classifications, as well as the sensitivity and specificity. A *p*-value ≤ 0.05 was deemed significant in all of the hypothesis tests. Patients with missing data for the specific variables under consideration in a statistical procedure were removed (but only for that procedure). All the inferential procedures were performed with R software version 4.3.1.

## 5. Conclusions

In this study, we assessed the MMR status in an EC series using three techniques: IHC, PCR, and genomic analysis (NGS plus MLPA). Although acceptable concordance was observed, there were certain discrepancies between the three testing approaches, mainly associated with the dMMR subgroup. IHC had a better correlation to MMR genomic status than to MSI status using PCR. Considering that immunotherapy appears to be an effective treatment for dMMR, further specific studies are needed to establish solid conclusions regarding the best MMR assessment technique in EC as a therapy response marker.

## Figures and Tables

**Table 1:** Overall comparisons between techniques: (**a**) MMR via genomic status and IHC, (**b**) MSI via PCR and MMR status via IHC, and (**c**) MMR genomic status and MSI via PCR.

(**a**)					
		**MMR Genomic Status**			
		**Altered**	**No**		**Total**
**IHC**	**dMMR**	46	21		67
	**pMMR**	9	48		57
	**Total**	55	69		124
(**b**)					
		**PCR**			
		**MSI-H**	**MSI-L**	**MSS**	**Total**
**IHC**	**dMMR**	22	7	34	63
	**pMMR**	3	3	45	51
	**Total**	25	10	79	114
(**c**)					
		**PCR**			
		**MSI-H**	**MSI-L**	**MSS**	**Total**
**MMR genomic status**	**Altered**	24	7	18	49
	**No**	1	3	59	63
	**Total**	25	10	77	112

MMR, mismatch repair; IHC, immunohistochemistry; MSI, microsatellite instability; PCR, polymerase chain reaction; dMMR, mismatch repair deficient; pMMR, mismatch repair proficient, MSI-L, microsatellite instability—low; MSI-H, microsatellite instability—high; MSS, microsatellite stable.

**Table 2:** Overall comparisons.

Reference Variable	Response Variable	Sensibility (%)	Specificity (%)	Total Proportion of Correct Classifications (%)
IHC	Genomic status	83.6	69.6	75.8
	PCR (MSS + MSI-L/MSI-H)	88.0	53.9	61.4
	PCR (MSS/MSI-L + MSI-H)	82.9	57.0	64.9
Genomic status	IHC	68.7	84.2	75.8
	PCR (MSS + MSI-L/MSI-H)	96.0	71.3	76.8
	PCR (MSS/MSI-L + MSI-H)	88.6	76.6	80.4

IHC, immunohistochemistry; PCR, polymerase chain reaction; MSI-L, microsatellite instability—low; MSI-H, microsatellite instability—high; MSS, microsatellite stable.

## Data Availability

The datasets are available from the corresponding authors upon request.

## Supplementary Materials

The supporting information can be downloaded at: https://www.mdpi.com/article/10.3390/ijms241914468/s1.
