# Supplementary material for: Comparison of Methods for Testing Mismatch Repair Status in Endometrial Cancer
Source: Int J Mol Sci. 2023 Sep 23;24(19):14468. doi: 10.3390/ijms241914468 (PMC10572657; doi:10.3390/ijms241914468)
Supplement: Supplementary file 1 [file ijms-24-14468-s001.zip › Supplementary Table S1.pdf]

Supplementary Table S1. Microsatellite instability detailed descriptive pattern based on Promega 1.2 results (n=114).

N (% of total)	BAT-25	BAT-26	NR-21	NR-24	MONO-27
**Stable**	94 (82.5)	91 (79.8)	103 (90.4)	102 (89.5)	88 (77.2)
**Unstable**	20 (17.5)	23 (20.2)	11 (9.6)	12 (10.5)	26 (22.8)

Twelve cases (9.5% of 126) had no available data and were therefore not considered for the descriptive calculations.
