# Supplementary material for: Comparison of Methods for Testing Mismatch Repair Status in Endometrial Cancer
Source: Int J Mol Sci. 2023 Sep 23;24(19):14468. doi: 10.3390/ijms241914468 (PMC10572657; doi:10.3390/ijms241914468)
Supplement: Supplementary file 1 [file ijms-24-14468-s001.zip › Supplementary Table S2.pdf]

Supplementary Table S2. Genomic status results based on next-generation sequencing and methylation-specific multiplex ligation-dependent probe amplification (n=124).

**N (% of total)**	***MLH1* (mut/met)**	***MLH1* (only met)**	***MLH1* (only mut)**	***PMS2 (mut)***	***MSH2 (mut)***	***MSH6 (mut)***	**Double alteration**
**Altered**	36 (29.0)	28 (22.6)	4 (3.2)	8 (6.5)	7 (5.6)	18 (14.5)	6 (4.8)

Two cases (1.6% of 126) had no available data and were therefore not considered for the descriptive calculations.
Mut, mutated; met, methylated.
