# Supplementary material for: Comparison of Methods for Testing Mismatch Repair Status in Endometrial Cancer
Source: Int J Mol Sci. 2023 Sep 23;24(19):14468. doi: 10.3390/ijms241914468 (PMC10572657; doi:10.3390/ijms241914468)
Supplement: Supplementary file 1 [file ijms-24-14468-s001.zip › Supplementary Table S3.pdf]

Suppl. Table S3. Detailed comparisons between MMR genomic status and IHC.

	MLH1		PMS2		MSH2		MSH6		MLH1/PMS2		MSH2/MSH6	
	Alt	No	Alt	No	Alt	No	Alt	No	Alt	No	Alt	No
Lost	28	10	28	10	3	6	7	7	32	19	10	6
Pres	7	77	7	77	4	110	11	98	6	65	14	93
**Correct classification proportion (%)**	86.1		65.0		91.9		85.4		79.5		83.7	
**Correlation (%)**	0.7		0.3		0.3		0.4		0.6		0.4	
**p**	4.9e-13		0.00661		0.00833		0.00083		1.3e-10		5.0e-5	

Alt, Altered; Pres, preserved. p-values calculated using Fisher’s exact independence test.
