# Supplementary material for: Comparison of Methods for Testing Mismatch Repair Status in Endometrial Cancer
Source: Int J Mol Sci. 2023 Sep 23;24(19):14468. doi: 10.3390/ijms241914468 (PMC10572657; doi:10.3390/ijms241914468)
Supplement: Supplementary file 1 [file ijms-24-14468-s001.zip › Supplementary Table S4.pdf]

Suppl Table S4. Panels for detailed comparisons between MMR status by IHC and PCR: a) classical and b) alternative groups.

a)

	**MLH1**		**PMS2**		**MSH2**		**MSH6**		**MLH1/PMS2**		**MSH2/MSH6**		
	Pres	Lost	Pres	Lost	Pres	Lost	Pres	Lost	Pres	Lost	Pres	Lost	Total
**MSS + MSI-L**	71	18	62	27	82	7	77	12	60	29	76	13	89
**MSI-H**	7	18	5	20	24	1	23	2	5	20	23	2	25
**Total**	78	36	67	47	106	8	100	14	65	49	99	15	114
**Coincidences proportion (%)**	78.1		71.9		72.8		69.3		70.2		68.4		
**Correlation (%)**	46.1		41.7		-6.3		-6.9		39.6		-8.1		
**p-value**	2.652e-06		1.531e-05		0.6832		0.7313		2.843e-05		0.5164		

b)

	MLH1		PMS2		MSH2		MSH6		MLH1/PMS2		MSH2/MSH6		
	Pres	Lost	Pres	Lost	Pres	Lost	Pres	Lost	Pres	Lost	Pres	Lost	Total
**MSS**	65	14	55	24	72	7	70	9	54	25	69	10	79
**MSI-L + MSI-H**	13	22	12	23	34	1	30	5	11	24	30	5	35
**Total**	78	36	67	47	106	8	100	14	65	49	99	15	114
**Coincidences proportion (%)**	76.3		68.4		64.0		65.8		68.4		64.9		
**Correlation (%)**	44.8		33.1		-10.8		4.1		34.4		2.2		
**p-value**	5.399e-06		0.00081		0.4314		0.7587		0.00040		0.773		

MSI-L, microsatellite instability - low; MSI-H, microsatellite instability - high; MSS, microsatellite stable; Pres, preserved. p-values calculated using Fisher's exact independence test.
