# Supplementary material for: Comparison of Methods for Testing Mismatch Repair Status in Endometrial Cancer
Source: Int J Mol Sci. 2023 Sep 23;24(19):14468. doi: 10.3390/ijms241914468 (PMC10572657; doi:10.3390/ijms241914468)
Supplement: Supplementary file 1 [file ijms-24-14468-s001.zip › Supplementary Table S5.pdf]

Suppl Table S5. Panels for detailed comparisons between MMR genomic status and MSI by PCR: a) classical and b) alternative groups.

a)

	**MLH1**		**PMS2**		**MSH2**		**MSH6**		
	No	Alt	No	Alt	No	Alt	No	Alt	Total
**MSS**	69	8	74	3	75	2	69	8	77
**MSI-L+MSI-H**	10	24	29	5	32	2	27	7	34
**Total**	79	32	103	8	107	4	96	15	111
**Coincidences proportion (%)**	83.8		71.2		69.4		68.5		
**Correlation (%)**	61.3		19.3		8.1		13.8		
**p-value**	3.859e-10		0.05599		0.5847		0.2262		

b)

	MLH1		PMS2		MSH2		MSH6		
	No	Alt	No	Alt	No	Alt	No	Alt	Total
**MSS + MSI-L**	75	12	84	3	85	2	75	12	87
**MSI-H**	4	20	19	5	22	2	21	3	24
**Total**	79	32	103	8	107	4	96	15	111
**Coincidences Proportion (%)**	85.6		80.2		78.4		70.3		
**Correlation (%)**	63.2		27.7		13.3		-1.6		
**p-value**	2.594e-10		0.01141		0.2036		1		

MSI-L, microsatellite instability - low; MSI-H, microsatellite instability - high; MSS, microsatellite stable.

p-values calculated using Fisher’s exact independence test.
